# Successful treatment of Hori nevus with a 755 nm ps alexandrite laser with a diffractive lens array

**DOI:** 10.1016/j.jdcr.2025.11.002

**Published:** 2025-11-07

**Authors:** Kentaro Ishii, Jun Omatsu, Hideki Shimura, Koya Sonoda

**Affiliations:** a Setagaya Sonoda Dermatology Clinic, Setagaya-ku, Tokyo, Japan; b Department of Dermatology, University of Tokyo Graduate School of Medicine, Bunkyo-ku, Tokyo, Japan; c Shimura Skin Clinic, Niigata, Niigata, Japan

**Keywords:** acquired bilateral nevus of Ota-like macules (ABNOM), diffractive lens array, fractional laser, Hori nevus, picosecond alexandrite laser

## Introduction

Hori nevus, also known as acquired bilateral nevus of Ota-like macules (ABNOM), is an acquired dermal melanocytosis that primarily affects Asian women and is characterized by scattered brown-to-gray patches on the cheeks. While various Q-switched and picosecond lasers have demonstrated efficacy in treating this condition, post-inflammatory hyperpigmentation remains a significant challenge, particularly in patients with darker skin types.

A 755-nm picosecond alexandrite laser (PSAL) equipped with a diffractive lens array (DLA) offers a fractional approach, concentrating high energy into microbeams surrounded by a low-fluence background. This technology is effective for skin rejuvenation and photoaging with minimal downtime, but its efficacy for deep dermal pigmentary lesions like Hori's nevus is not yet well established.

Herein, we report 2 cases suggesting that this fractional PSAL with DLA may provide an effective and safe treatment option for Hori nevus, with the potential for greater efficacy and shorter downtime than conventional laser therapies.

## Case report

Two healthy Asian women with Fitzpatrick skin type III presented with scattered brown-to-slate-gray patches in the zygomatic region, clinically diagnosed as Hori nevus. Treatments were performed using a 755-nm picosecond alexandrite laser (PicoSure, Cynosure Lutronic Inc). Standardized facial imaging was performed using the re-Beau 2 system (JMEC Co, Ltd) for both patients.

### Patient 1

**Fig 1:**
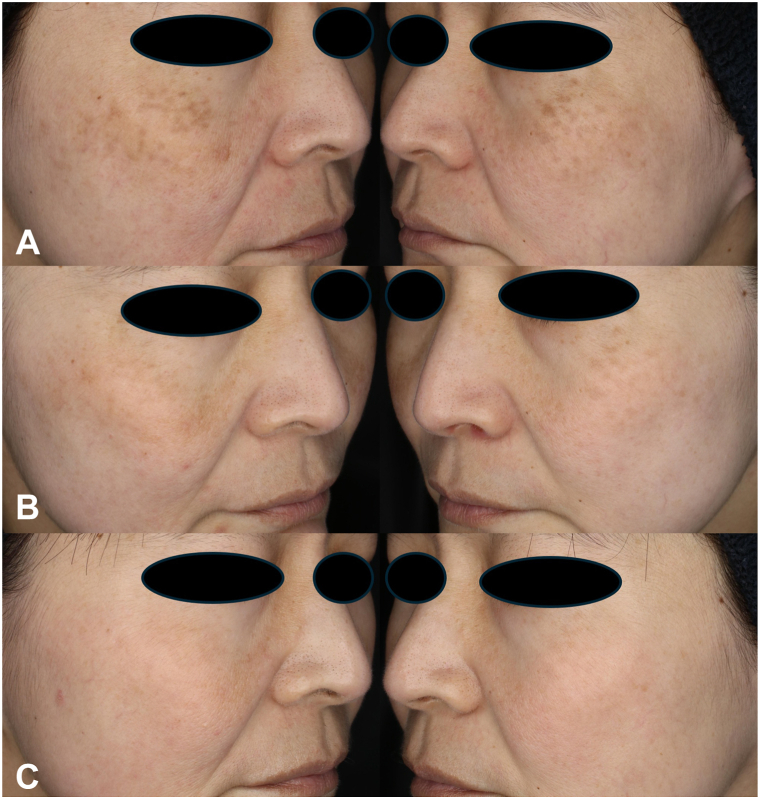
**A,** Baseline. **B,** Three months after treatment with a 6-mm spot size with DLA on the right cheek and a 3-mm spot size without DLA on the left cheek. **C,** Eight months after an additional single treatment with a 6-mm spot size with DLA on both cheeks.

A 46-year-old woman underwent a single laser treatment at another clinic 3 months before presented for further management (details unknown). At baseline (Fig 1, *A*), the initial session at our clinic was performed using a 3-mm spot size (550 ps, 2.33 J/cm^2^, 5 Hz, single pass) on the left side and a 6-mm spot size with DLA (550 ps, 0.57 J/cm^2^, 5 Hz, 6 passes) on the right side. The 3 mm conventional setting was chosen to replicate parameters commonly used for dermal pigment lesions, whereas the 6 mm DLA mode was constrained by its fixed array optics, which limit fluence to <0.6 J/cm^2^ and require multiple passes for full coverage. Post-treatment erythema and inflammation were less pronounced on the DLA-treated side. At the 3-month follow-up (Fig 1, *B*), greater improvement was observed on the DLA-treated side. A second session using a 6-mm spot size with DLA on both sides led to complete resolution of pigmentation by the 8-month follow-up (Fig 1, *C*). No post-inflammatory hyperpigmentation (PIH), scarring, or other adverse events occurred, and erythema resolved within 3 days. The patient remained recurrence-free and lesion-clear at the 12-month follow-up.

### Patient 2

**Fig 2:**
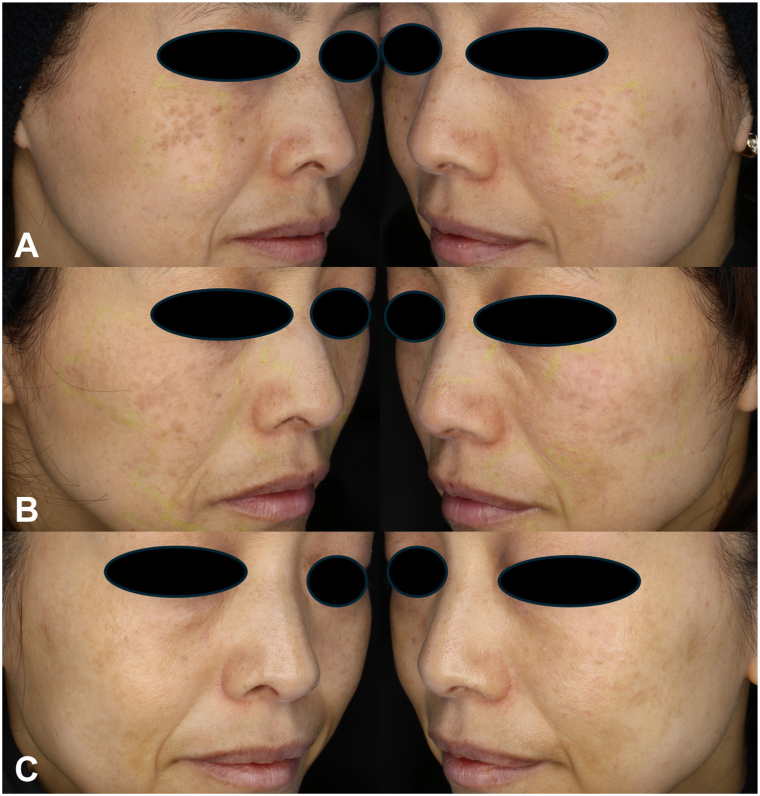
**A,** Baseline. **B,** Three months after treatment with a 3.6-mm spot size without DLA on the right cheek and a 6-mm spot size with DLA on the left cheek. **C,** Ten months after 2 additional treatments with a 6-mm spot size with DLA on both cheeks.

A 50-year-old woman with no prior laser treatment history presented with bilateral brownish macules (Fig 2, *A*). At baseline, the initial session was performed using a 3.6-mm spot size (550 ps, 1.62 J/cm^2^, 5 Hz, single pass) on the right side and a 6-mm spot size with DLA (550 ps, 0.57 J/cm^2^, 5 Hz, 6 passes) on the left side. Post-treatment erythema and inflammation were again milder on the DLA-treated side. Post-treatment erythema and inflammation were again milder on the DLA-treated side. At 3 months, a second session using the same parameters was performed; the third and fourth sessions (months 6 and 9, respectively) employed a 6 mm spot size with DLA on both sides, resulting in near-complete clearance by the 10-month follow-up (Fig 2, *C*). Transient erythema subsided within several days after each session, and no PIH, scarring, or recurrence was observed during the 12-month follow-up.

## Discussion

Hori's nevus, also known as ABNOM, is an acquired dermal melanocytosis primarily affecting Asian women. The lesions are characterized by scattered brown-to-gray patches on the cheeks^1^ and histologically by clusters of melanocytes in the superficial dermis, particularly around blood vessels.

Although various lasers have shown efficacy, PIH remains problematic, especially in darker skin types. Yu et al conducted a split-face study comparing Q-switched alexandrite laser (QSAL) and PSAL, reporting superior efficacy of PSAL with less pain and fewer hyperpigmentation issues.^2^

DLA technology concentrates about 70% of the total energy into less than 10% of the treated area, leaving the remaining 30% as a low-fluence background.^3^ It is effective with minimal downtime for skin rejuvenation and photoaging.

Among picosecond laser systems, the 755-nm alexandrite laser with a DLA creates laser-induced optical breakdown (LIOB) mainly in the epidermis, triggering indirect dermal remodelling rather than direct ablation.^4^ Because DLA focal depth is 150-200 μm, effects on deeper pigment remain unclear. Tanghetti et al noted punctate dermal haemorrhage in low-melanin skin, suggesting hemoglobin can act as an alternative chromophore.^5^ These findings imply that DLA microbeams might reach superficial dermal melanocyte nests in Hori’s nevus, generate LIOB in situ, and perhaps explain the clearance we observed. In this study, different parameters were selected to reflect the fractional energy distribution and epidermal LIOB characteristics of the DLA optic described in prior studies, and to ensure even coverage in clinical practice. Multiple passes were therefore used with the DLA mode in our protocol. These parameter choices were informed by prior reports of fractional PSAL for photoaging^3^^,^^4^ and by our clinical experience with Asian skin types. In our system, the DLA handpiece operates with a 6-mm treatment field and a relatively low per-pulse fluence compared with conventional small-spot optics, necessitating multiple passes for uniform coverage. A limitation is that multiple passes are required to treat the entire area, as the laser concentrates energy into tiny “points”, potentially increasing the risk of scarring if spots overlap excessively.

Further research is necessary to optimize parameters such as spot size, energy level, pass count, and treatment interval. Nevertheless, these cases suggest that fractional PSAL with DLA can achieve effective pigment removal with minimal downtime, indicating its promise as a treatment option for Hori's nevus.

## Conflicts of interest

None disclosed.
